# An insidious internal hernia 30 years after jejunoileal bypass: a case report

**DOI:** 10.1093/jscr/rjad665

**Published:** 2023-12-05

**Authors:** Ville A Palomäki, Saana Palomurto, Sari Venesmaa, Pirjo Käkelä

**Affiliations:** Department of Surgery, University of Eastern Finland and Kuopio University Hospital, Kuopio, Finland; Department of Surgery, University of Eastern Finland and Kuopio University Hospital, Kuopio, Finland; Department of Surgery, University of Eastern Finland and Kuopio University Hospital, Kuopio, Finland; Department of Surgery, University of Eastern Finland and Kuopio University Hospital, Kuopio, Finland

**Keywords:** jejunoileal bypass, internal hernia, CT scan, whirl sign

## Abstract

Jejunoileal bypass (JIB) was an early bariatric procedure that involved bypassing most of the small bowel resulting in malabsorption and weight loss. Due to serious complications associated with the procedure, JIB was largely discontinued by the mid-1980s. We report the case of a 77-year-old woman with a history of JIB 31 years earlier. In 2022, she was hospitalized for acute abdominal pain. A computed tomography (CT) scan revealed a suspicion of internal hernia (IH) with a typical swirl sign. Due to the quick relief of symptoms an emergency surgery was not considered at the time. Nevertheless, a subsequent operation revealed a large mesenteric defect, adhesions and 100 cm of effective small bowel left. Although the procedure is no longer performed, some patients with JIB are still alive and develop late complications. To our knowledge, this is the first case report describing an IH in a patient who has undergone JIB.

## Introduction

Jejunoileal bypass (JIB) was a surgical procedure used to treat obesity in the 1970s and 1980s, prior to modern bariatric operations, such as Roux-en-Y gastric bypass (RYGB) [1, 2]. The technique involved anastomosing the jejunum and ileum, resulting in an effective small bowel length of 45 cm left for absorption [1, 3].

JIB typically resulted in weight loss of 45–64 kg or a mean body mass index (BMI) reduction from 48 to 32 over a few years [1, 3]. Many patients experienced nutritional deficiencies or metabolic issues, such as malabsorption, diarrhea, electrolyte imbalances, gallstones, kidney stones, abdominal pain, bloating, or even liver failure and death [4]. Due to the uncertainty of the actual benefits of the procedure in regards of risks, JIB was largely abandoned by the mid-1980s [5].

Although complications related to short bowel length were common after JIB, cases of intestinal obstruction due to adhesions or internal herniation are rarely reported. We present here a case report of a patient with small bowel obstruction due to internal hernia (IH) after 32 years of JIB.

## Case report

A 45-year-old female with a long-term history of obesity and a BMI of 56 (165 kg) was referred to bariatric surgery in 1990. At the time, she had no comorbidities other than musculoskeletal disorders. She had previously lost 15 kg with conservative means but regained the weight.

In 1991, a laparotomy and JIB were performed. According to the old surgical report, the jejunum was divided 25 cm from the ligament of Treitz and anastomosed to the ileum 25 cm proximal to the ileocecal valve. The patient was discharged with calcium and iron supplements. In follow-up, the patient’s weight decreased to 114 kg (BMI 42) and low hemoglobin values were measured. She had no electrolyte imbalances or other signs of serious malabsorption. The patient reported a maximum weight loss of 60 kg.

**Figure 1:**
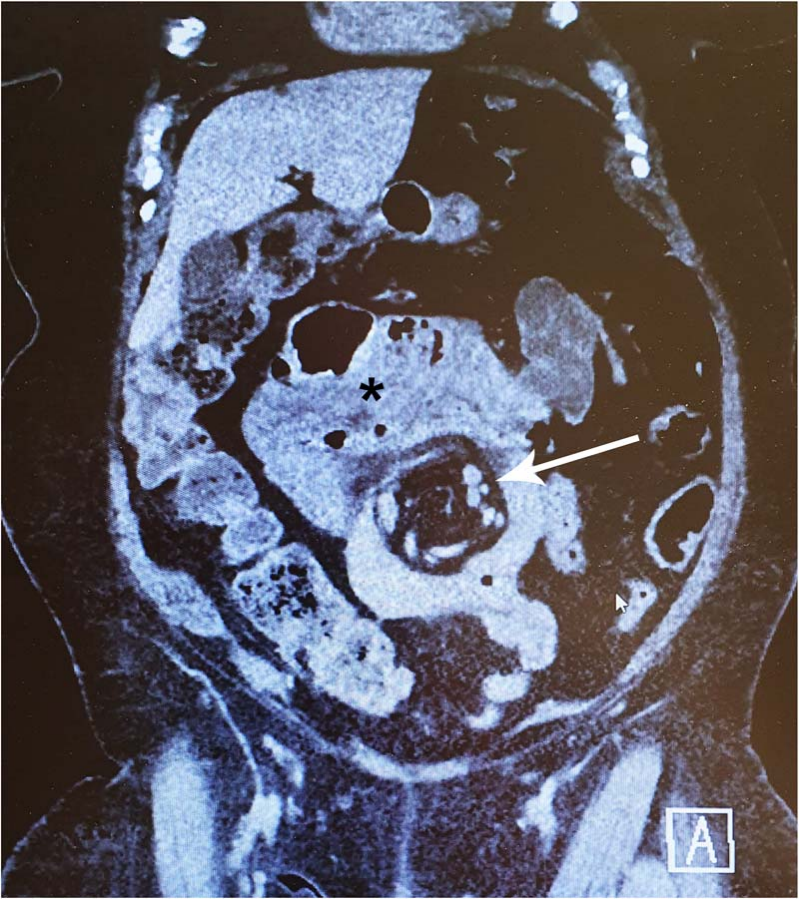
A 77-year-old woman with a history of jejunoileal bypass 31 years ago presented with intermittent colicky upper abdominal pain for 1–2 years. A rather painful episode led to hospital admission and CT scan, which showed a mesenteric swirl sign (arrow). Symptoms quickly subsided. Although she remained symptomless after that particular episode, an exploratory laparoscopy converted to laparotomy was performed 5 months later. This revealed a large mesenterial defect under the jejunoileal anastomosis. 390 cm of bypassed small bowel was found to be atrophied and packed with adhesions. This finding may be visible on CT scan (*).

In late 2022, this now 77-year-old patient came to the emergency ward with intense abdominal pain. She had been experiencing transient colicky epigastric pain infrequently for the past few years. At this time, her medical history included hypertension, musculoskeletal disorders, diverticulosis, and a deep vein thrombosis in 2015. She took a daily multivitamin supplement and additional zinc, vitamin C and D, and received vitamin B12 injections every 3 months. A computed tomography (CT) scan revealed a suspicion of IH with a typical swirl sign in the mesenteric vascular structures (Fig. 1). There were no signs of compromised bowel blood perfusion or dilatation in the bowels or stomach. At the time, the exact procedure the patient had undergone 31 years earlier was unclear. The symptoms resolved quickly, and the emergency operation was not necessary. At follow-up, the patient remained symptom-free. Archived medical records revealed that she had undergone a JIB in 1991. In agreement with the patient, we proceeded with exploratory laparoscopy.

Due to heavy adhesion formation, bowel anatomy could not be resolved during the laparoscopy. Therefore, a laparotomy was performed and a myriad of bowel adhesions was released, and a long remnant segment of atrophied small bowel was identified. A large mesenterial defect under the jejunoileal anastomosis was identified and closed with sutures. In contrast to the 30-year-old surgical report, the patient had 100 cm of remaining effective small bowel left and she appeared to be able to manage well with that amount of bowel. A comprehensive set of laboratory findings were within normal range at 1 month postoperatively (Table 1).

**Table 1:** Analysis of the blood samples.

Variable	Result	Reference	Unit
Hemoglobin	122	117–155	g/l
WBC	5.7	3.4–8.2	E9/l
E-MCV	89	82–98	fl
E-MCH	29	27–33	pg
Thrombocytes	198	150–360	E9/l
Ferritin	89	13–150	μg/l
Transferrin receptor	3.1	1.7–4.1	mg/l
APTT	25	23–33	s
INR	1.0	0.9–1.2	
Fasting glucose	6.7	4–6	mmol/l
Albumin	37	34–45	g/l
Prealbumin	0.22	0.18–0.39	g/l
Sodium	140	137–144	mmol/l
Potassium	3.9	3.4–4.7	mmol/l
Calcium-ion	1.22	1.16–1.3	mmol/l
Phosphate	1.01	0.76–1.41	mmol/l
Magnesium	0.74	0.71–0.94	mmol/l
Copper	17.3	12.6–24.4	μmol/l
Zink	10.4	9–22	μmol/l
Selenium	1.34	0.64–1.52	μmol/l
Creatinine	65	50–90	μmol/l
Creatine kinase	58	35–210	U/l
Aspartate aminotransferase	20	15–35	U/l
Alanine aminotransferase	19	<35	U/l
Alkaline phosphatase	57	35–105	U/l
Bilirubin	7	5–25	μmol/l
Conjugated bilirubin	3	1–5	μmol/l
Cholesterol	4.5	<5	mmol/l
HDL-cholesterol	1.31	>1.2	mmol/l
LDL-cholesterol	2.8	<3	mmol/l
Triglycerides	1.14	<1	mmol/l
Folate	34.1	>7	nmol/l
Vitamin B1	223	60–230	nmol/l
Vitamin B12	133	>35	pmol/l
25-hydroxyvitamin D	69	>50	nmol/l

Abbreviations: WBC: white blood cell count; MCV: mean corpuscular volume; MCH: mean corpuscular hemoglobin; APTT: activated partial thromboplastin time; INR: international normalized ratio of prothrombin time; HDL: high-density lipoprotein; LDL: low-density lipoprotein.

## Discussion

JIB is a long-discontinued bariatric surgery procedure. Some patients who underwent it are still alive, and late complications have been reported [6].

Similarly, to modern bypass operations, rerouting the small bowel continuity in JIB created an arbitrary mesenteric defect under the anastomosis. The 31-year-old surgical reports did not mention whether the defect was closed. Nowadays, it is advocated that all mesenterial defects should be closed after bariatric operations to prevent internal herniation [7, 8]. Proper defect closure reduces the incidence of IH to 1% [7]. IH may require an urgent surgical intervention [9]. Surgical findings may include edematous swelling of the mesentery and from slight color change up to incarceration of the bowel [10]. According to our experience, IH may sometimes resolve before emergency surgery spontaneously. In these cases, indirect signs of recent herniation, such as erythema and swelling of the bowel or the edges of the hernia defect, may be observed.

We found no case reports of IH occurring after JIB. Instead, the phenomenon is well-recognized after RYGB and may present with acute onset of colicky epigastric pain, sometimes even radiating to the back, and the symptoms may worsen with eating and be accompanied by vomiting due to bowel obstruction [7, 9]. Nevertheless, the symptoms are often vague and nonspecific, which can make diagnosis challenging and result in delays [11]. The range of consequences of an IH may vary from episodic abdominal pain to bowel strangulation through the defect [7, 12]. A CT scan is helpful in diagnostics but may appear normal in up to 30% of cases and does not exclude the IH [12–14]. The best single finding on a CT scan that suggests IH is a “whirl sign”, in which the mesenteric vessels or fat appear in a swirling pattern [15]. Other suggestive findings include small bowel obstruction or clustered loops, any part of the bowel other than the duodenum behind the superior mesenteric artery, right-sided, or superior to transverse colon location of jejunojejunostomy or mushroom-shaped mesenteric root [14, 15].

In conclusion, it is likely that the patient had an intermittent IH that caused her colicky abdominal pain. This is supported by the presence of a clear mesenteric swirl sign on the CT scan. However, we speculate that the herniation spontaneously resolved during her first hospital admission, which led to the relief of her symptoms. The exact nature of her previous bariatric operation was unclear at the time, as her old medical records were not available. The quick relief of her symptoms during the hospital admission led to the decision to refrain from emergency surgery.

## Data Availability

All data supporting the case report are represented within the article.
